# Neutrophils: Their Role in Innate and Adaptive Immunity 2017

**DOI:** 10.1155/2017/9748345

**Published:** 2017-11-07

**Authors:** Carlos Rosales, Clifford A. Lowell, Michael Schnoor, Eileen Uribe-Querol

**Affiliations:** ^1^Instituto de Investigaciones Biomédicas, Universidad Nacional Autónoma de México, 04510 Ciudad de México, Mexico; ^2^Department of Laboratory Medicine, University of California, San Francisco, CA 94143, USA; ^3^Department of Molecular Biomedicine, CINVESTAV-IPN, 07360 Ciudad de México, Mexico; ^4^Facultad de Odontología, Universidad Nacional Autónoma de México, 04510 Ciudad de México, Mexico

Neutrophils have long been regarded as the first line of defense against infection and one of the main cell types involved in initiation of the inflammatory response. It is generally accepted that the innate immunity functions of neutrophils are mainly mediated by phagocytosis, release of granules, and formation of neutrophil extracellular traps (NETs). In recent years, however, accumulating evidence has shown that neutrophils possess greater functional diversity than previously appreciated. Thus, the classical view of neutrophils as simple cytotoxic leukocytes against pathogens is currently being revisited. Neutrophils display an array of biological functions important for both innate and adaptive immune responses. Neutrophils can produce many cytokines and chemokines upon stimulation, and in this way, they can interact with endothelial cells, dendritic cells, macrophages, natural killer cells, T lymphocytes, and B lymphocytes. Through all these interactions, neutrophils can either activate or downregulate both innate and adaptive immunity. The novel functions of neutrophils reveal that these cells have unanticipated roles in homeostasis, as well as in several diseases such as atherosclerosis, stroke, chronic obstructive pulmonary diseases, and cancer.

To continue elucidating the complex role of neutrophils in infection, inflammation, and immunity, this second special issue has brought together original and review articles that will help us to better understand the complex and fascinating neutrophil biology.

As mentioned before, the primary role of neutrophils is the clearance of extracellular pathogens, through phagocytosis, release of a broad array of effector molecules, and the production of extracellular traps. However, some pathogens have also the capacity to overcome neutrophil-mediated host defense mechanisms and establish infections leading to disease. One such pathogen is the bacteria *Staphylococcus aureus*, which can block chemotaxis and phagocytosis, thus evading killing by neutrophils. In addition, *S. aureus can* survive within neutrophils and promote neutrophil cytolysis, causing the release of molecules that promote local inflammation. The article by T.-S. Teng and colleagues describes the mechanisms by which neutrophils kill extracellular pathogens and how pathogens evade neutrophil defense mechanisms. They also discuss ideas that might be useful for the development of novel therapies against infections caused by antibiotic-resistant pathogens.

Similarly, the role of NETs is primarily to entrap extracellular microbes and, in this way, to keep an early infection localized. Yet, besides microorganisms, other stimuli can also activate neutrophils to produce NETs. The article by B. Rada summarizes the recently described ability of different microcrystals to induce NET formation. Microcrystals are insoluble crystals with a size of 1–100 micrometers that can irritate phagocytes including neutrophils and typically trigger an inflammatory response. The effect on neutrophils by microcrystals in adjuvant and by microcrystals associated with diseases such as gout, atherosclerosis, and silicosis is discussed.

Neutrophils play an essential role during an inflammatory response. They are rapidly mobilized from the circulation into damaged tissues. The blood supply of neutrophils is at the same time replenished by a rapid recruitment of neutrophils from the bone marrow to the vasculature. A great deal is known about the mechanism for neutrophil migration into tissues. However, there is very little information about the molecular signals that regulate the entry of neutrophils into the circulation. In an attempt to learn more about this process, the article by C. Zuñiga-Traslaviña and colleagues describes a zebrafish model, to assess the role of CXC-chemokines and CXC-receptor 2, in neutrophil migration into the blood circulation after injury. They found that the CXCL8b/CXCR2 axis is an important regulator of neutrophil entry into the bloodstream. On the other hand, when neutrophils arrive at sites of inflammation, they release various cytokines. The signaling machinery required for production of inflammatory and immunomodulatory cytokines and for extending the life of neutrophils at inflamed sites is poorly known. Thus, this is another area of intense study in the neutrophil field. The article by T. Ear and colleagues tells us about the constitutive expression of various Src family kinase isoforms and of spleen tyrosine kinase (Syk) in neutrophils and how the inhibition of these tyrosine kinases selectively blocks inflammatory cytokine production by acting posttranscriptionally. In contrast, delayed apoptosis seems to be independent of these kinases. These findings have implications for the future identification of potential molecular targets that could be useful in therapeutic intervention of chronic inflammatory conditions.

The involvement of neutrophils in several inflammatory pathologies is being recognized more and more, with the growing understanding of the proinflammatory and immunomodulatory properties of these cells. In some conditions, such as stroke and cancer, the numbers of immune cells can significantly predict the clinical outcome of the disease. In particular, the neutrophil-to-lymphocyte ratio was shown to predict hemorrhagic transformation and the clinical outcome of stroke. However, the immunological mechanisms underlying these effects are poorly understood. In the article by J. Ruhnau and colleagues, the role of neutrophils in brain ischemia is discussed. Neutrophils are the first cells to invade injured tissue after focal brain ischemia. In these conditions, they can enhance tissue damage and even promote more ischemic injury by inducing thrombus formation. Yet, neutrophils are also beneficial because they are involved in triggering the removal of cell debris and are essential for defense against bacterial infections. Thus, therapeutic interventions that target neutrophils to prevent stroke should preserve their functions outside the central nervous system.

In other pathologies, neutrophils also play an essential role. For example, respiratory diseases such as asthma and chronic obstructive pulmonary disease (COPD) are characterized by an excessive infiltration of neutrophils. It is generally accepted that subsequent activation of these neutrophils promotes production of reactive oxygen species and release of proteases resulting in tissue damage and alveolar airspace enlargement. The article by J. Liu and colleagues reviews the role of neutrophils in respiratory diseases, describing recent studies on mechanisms for neutrophil trafficking, activation, and cell death. The studies on neutrophil function with isolated cells do not provide a complete picture because cells are taken from the inflammatory environment of the disease. Animal models play an important role in studying the underlying mechanisms of respiratory diseases such as COPD as they address questions involving integrated whole body responses. The article by G. Huang and colleagues presents a review of the current animal models of COPD, focusing on their advantages and disadvantages on immune responses and neutrophilic inflammation. Finally, the article by C. K. Mårdh and colleagues discusses novel therapeutic approaches for respiratory diseases that target neutrophil function. They describe how targeting the chemokine receptors CXCR2 and CXCR1 could be regulated during neutrophil trafficking and how targeting the enzyme PI3K could modulate neutrophil function. Also, they explain how protease inhibitors that target matrix metalloproteinases and neutrophil serine proteases could prevent excessive tissue damage.

Together, these articles provide a sample of the multiple and complex functions of neutrophils for fighting infections and for controlling immunity. They also underline the relevant role of neutrophils in pathological conditions and provide guidance for future research on the cell biology of these fascinating leukocytes.

*Carlos Rosales* *Clifford A. Lowell* *Michael Schnoor* *Eileen Uribe-Querol*

